# Brain microvascular disease and functional network connectivity—a call for a stage-based approach

**DOI:** 10.1093/braincomms/fcad135

**Published:** 2023-04-24

**Authors:** Stefanie Schreiber, Philipp Arndt, Sven G Meuth, Alexander Dityatev, Hendrik Mattern

**Affiliations:** Department of Neurology, Otto von Guericke University Magdeburg, Magdeburg 39120, Germany; German Center for Neurodegenerative Diseases (DZNE) within the Helmholtz Association, Magdeburg 39120, Germany; Center for Behavioral Brain Sciences (CBBS), Magdeburg 39120, Germany; Medical Faculty, Otto von Guericke University, Magdeburg 39120, Germany; Center for Intervention and Research on adaptive and maladaptive brain Circuits underlying mental health (C-I-R-C), Jena 07737-Magdeburg-Halle 06108, Germany; Department of Neurology, Otto von Guericke University Magdeburg, Magdeburg 39120, Germany; German Center for Neurodegenerative Diseases (DZNE) within the Helmholtz Association, Magdeburg 39120, Germany; Department of Neurology, Heinrich Heine University Düsseldorf, Düsseldorf 40225, Germany; German Center for Neurodegenerative Diseases (DZNE) within the Helmholtz Association, Magdeburg 39120, Germany; Center for Behavioral Brain Sciences (CBBS), Magdeburg 39120, Germany; Medical Faculty, Otto von Guericke University, Magdeburg 39120, Germany; German Center for Neurodegenerative Diseases (DZNE) within the Helmholtz Association, Magdeburg 39120, Germany; Center for Behavioral Brain Sciences (CBBS), Magdeburg 39120, Germany; Department of Biomedical Magnetic Resonance, Faculty of Natural Sciences, Otto von Guericke University Magdeburg, Magdeburg 39120, Germany

## Abstract

This scientific commentary refers to ‘Functional connectivity in older adults—the effect of cerebral small vessel disease’ by Drenth *et al.* (https://doi.org/10.1093/braincomms/fcad126).

This scientific commentary refers to ‘Functional connectivity in older adults—the effect of cerebral small vessel disease’ by Drenth *et al.* (https://doi.org/10.1093/braincomms/fcad126).

Experimentally, the link between health and integrity of the cerebral microvasculature and neuronal function is well established, emphasizing that microvascular cells, neurons and their synaptic connections form a functionally integrated network.^1^ The capacity for repair in response to microvascular injury, e.g. due to vascular risk factors such as arterial hypertension, cumulatively fails with increasing age, determines microvascular ageing and contributes to reduced synaptic/neuronal plasticity and impaired cognitive function.^2^

Studying functional brain connectivity by applying functional magnetic resonance imaging (fMRI) and relating it to structural pathologies of cerebral small vessel disease (CSVD) defined through STandards for ReportIng Vascular changes on nEuroimaging (STRIVE) ^3^ are currently the most feasible approach to investigate the association between synaptic and neuronal function and brain microvascular disease in a translational setting in humans. However, contrary to the experimental evidence, results of human studies on the association between functional connectivity and CSVD are heterogeneous, reporting increased, unaltered or decreased network activity—mainly affecting the default mode, dorsal attention and frontoparietal control network—in brain microvascular disease.^4^

To this end, Drenth *et al*.^5^ analysed 10 large-scale resting-state functional brain networks, as well as two global network markers for the whole brain, in a large well-characterized cohort of adults in the general population aged 75 years and older, and not having dementia with arterial hypertension, to address how ageing and CSVD burden, determined through a composite score of non-haemorrhagic and haemorrhagic STRIVE markers and grey matter atrophy, impact network activity.

This meticulous work did not reveal any significant direct or interacting effect of ageing and CSVD burden on functional connectivity.

We will briefly describe some aspects that could have affected the null results.

Selected functional networks have been shown to exhibit a non-linear, stage-dependent dynamic pattern in ageing and along the Alzheimer’s disease continuum, e.g. following the pattern of an inverted U shape with network hyperactivation in subjects with mild cognitive impairment compared to network hypo- or deactivation in cognitively normal and demented subjects.^6^ Different functional connectivity patterns within patient cohorts with non-uniform cognitive phenotypes might therefore obscure effects at the group level. The cohort investigated by Drenth *et al*. indeed consisted of a mix of cognitively normal and mildly cognitively impaired participants. Additionally, the microvascular endotype, i.e. CSVD severity, was heterogeneous. Given the mean age of around 80 years (SD of 4) in the participants without dementia, a comparably small number had moderate, and only few subjects displayed severe and long-standing CSVD, while a large proportion had probably rather mild and early CSVD. This is supported by the following aspects: (i) none of the cases had any history of stroke or transient ischemic attack; (ii) nearly 80% of the subjects had either no (ca. 40%) or low (ca. 40%) overall CSVD burden, while severe CSVD burden was existent in only 4% of the participants; (iii) mean systolic and diastolic blood pressure values were comparably well controlled (on average 145/81 mmHg); and (iv) only around two-thirds of the study population was already on long-term pharmacological blood pressure treatment despite the whole cohort suffering from arterial hypertension.

We have shown that rodents at early stages of chronic arterial hypertension and initial brain microvascular disease exhibit an energy-demanding hypermetabolic state accompanied by neuroinflammatory signatures of activated resident immune cells and peripheral immune cells leaking across the leaking blood–brain barrier (BBB).^7,8^ In comparison with normotensive control animals, ^18^F-fluorodeoxyglucose positron emission tomography (^18^F-FDG PET) in early chronic hypertensive rodents likewise revealed increased glucose metabolism, which largely declined at later chronic hypertensive stages.^7^ We discuss that the early ^18^F-FDG PET signal increase might mirror elevated glucose consumption of metabolically activated vascular cells, immune cells and synapses/neurons. These pathomechanisms could lead to an overall increased metabolic demand or could compensatorily lead to increased neuronal activity (e.g. to maintain cognitive function in the face of preexisting pathology), both of which would alter the fMRI signal towards increased activity. A recent meta-analysis focusing on resting-state fMRI in CSVD patients supports our experimental assumption showing increased network connectivity in early-stage disease (here defined through normal cognitive function), while decreased functional connectivity was particularly existent in cognitively impaired subjects in later disease stages, mirroring the non-linear functional network activity along the AD continuum.^9^

Disentanglement of the proposed non-linear functional network activity in brain microvascular disease, especially the detection of early increased functional connectivity, demands for better stratification of CSVD patients beyond clinical and cognitive phenotype and beyond the STRIVE MRI markers, which, despite being well-established, mainly depict (parenchymal) downstream pathologies. Instead, assessment and quantification of early brain microvascular dysfunction should include emerging MRI measures, such as vascular reactivity and pulsatility, BBB integrity, or measures of perivascular clearance.^10^ Patterns of functional network connectivity could prospectively add to these vascular markers additionally denoting CSVD initiation. While testing of targeted neurovascular unit treatments is primarily still in the preclinical phase, certain interventions potentially preserving microvascular maintenance before the occurrence of tissue damage can already be advised to patients in which initial microvascular disease is detected, underlining the clinical relevance to systematically implement methods for early microvascular dysfunction assessment. It is essential to educate affected subjects about the pivotal role of the protective effects of, e.g. adequate blood pressure and vascular risk factor control, regular mild to moderate exercise, sleep hygiene and chronic psychosocial stress reduction. We are convinced that future studies considering a kind of staging into early, advanced and later brain microvascular disease on brain network function will allow in-depth characterization of the functional unit between cerebral small vessels and synaptic/neuronal integrity in humans. That approach will not only help to overcome the so far largely heterogeneous results in the field but will also additionally aid in the establishment of new disease markers that will be prospectively implemented into STRIVE towards a new integrated classification of CSVD-related MRI markers.
